# An Overview of *Trypanosoma cruzi* Biology Through the Lens of Proteomics: A Review

**DOI:** 10.3390/pathogens14040337

**Published:** 2025-03-31

**Authors:** Jenny Telleria, Jaime A. Costales

**Affiliations:** 1 Institut de Recherche Pour le Développement (IRD), UMR Intertryp IRD-CIRAD, 34398 Montpellier, Cedex 5, France; jenny.telleria@ird.fr; 2 Centro de Investigación Para la Salud en América Latina, Pontificia Universidad Católica del Ecuador, Quito 170525, Ecuador

**Keywords:** parasite, proteome, metabolism, biology, life cycle, gene expression, parasite–host interaction

## Abstract

The protozoan parasite *Trypanosoma cruzi*, causative agent of Chagas disease, affects millions of people in endemic Latin American countries and beyond. In Latin America, Chagas disease is an important cause of death and disability, for which vaccines are lacking and improved treatment options are required. Additionally, the factors governing the development of a variety of clinical manifestations during Chagas disease, ranging from complete lack of symptoms to severe irreversible chronic organ damage (mainly cardiac or digestive), remain largely unknown. Much remains to be learned regarding the biology of *T. cruzi* in order to enhance our understanding of these lines of inquiry. In this context, proteomic methods have been leveraged to investigate different parasite strains, life-cycle forms, subcellular compartments, macromolecular complexes, signaling events and secreted molecules. The factors driving morphological transformation during the life cycle, the composition and functions of the parasite’s organelles and secreted molecules as well as the determinants of pathogenicity have been explored via proteomic methods, yielding insights into the fundamental processes behind the parasite biology and informing drug design and vaccine development. Importantly, the correlation between the wide genetic and phenotypic variability displayed by *T. cruzi* has been examined through proteomic methods as well. Here, we review the literature on *T. cruzi* proteomics and discuss it in the light of its limitations and in the context of the parasite’s genetic diversity.

## 1. Chagas Disease

Chagas disease is a chronic infection caused by the flagellated protozoan *Trypanosoma cruzi*, which is endemic to 21 countries in the Latin American continent. In recent years, due to human migration, Chagas disease has spread beyond the areas of endemic transmission as infected individuals have established residence in countries such as Canada and the United States, as well as European and Asian countries [1]. Currently, ~6 million people are infected and ~70 million live under risk of infection with *T. cruzi*. It is estimated that each year, the disease causes 12 thousand deaths, while 30 thousand new Chagas disease cases occur in the same period (https://www.paho.org/en/topics/chagas-disease, accessed on 19 March 2025).

The World Health Organization/Pan-American Health Organization (WHO/PAHO) has launched an initiative to implement comprehensive and sustainable actions which aimed to eliminate 30 communicable diseases and related conditions by 2030. Zoonotic diseases, such as Chagas disease, are included in the initiative (https://www.paho.org/en/elimination-initiative, accessed on 19 March 2025).

Chagas disease transmission takes place mainly through the activity hematophagous insects, commonly known as “kissing bugs”, which harbor *T. cruzi* in the digestive tract and transmit it by the stercorarian route. However, the disease can also be transmitted, though less frequently, by the congenital or oral routes, as well as via transfusion of *T. cruzi*-infected blood and blood products, organ transplantation and sporadically, during laboratory accidents [2].

Chagas disease progresses through an initial symptomatic or asymptomatic acute phase (lasting ~2 months) and a chronic phase. The clinical manifestations of the latter phase are divided into indeterminate (asymptomatic) form and a symptomatic chronic form (which includes cardiomyopathy and/or digestive pathology).

Currently, no effective vaccines for Chagas disease have been developed and only two drugs are approved for parasitological treatment of *T. cruzi* infection: benznidazole (BNZ) and nifurtimox (NFX). Both drugs have considerable side effects, which can range from hypersensitivity reactions and/or anorexia, at the beginning of treatment, to peripheral neuropathies or even psychosis at the end of treatment. A mutagenic and teratogenic effect has been demonstrated in experimental animals, which has not been evidenced in humans. Both drugs are contraindicated in pregnant women [3,4]. NFX acts on the parasite’s redox mechanism, while BNZ acts as a toxic agent [5]. Treatment is 100% successful in infants under age one; however, in older children and adults, effectiveness is only ~60% during the acute phase, while the effectiveness in the chronic phase is still a matter of debate [6,7]. Treatment failure may be related to the genetic diversity of the parasite and/or hosts [8].

## 2. Proteomics and *T. cruzi*

In 2005, as the product of the collaborative effort carried out by an international consortium, an initial version of the complete *T. cruzi* genome was published [9]. A year before, genomic databases for the parasite had been established and since, they have served as key resources in *T. cruzi* research [10]. Currently, this database is a bioinformatics resource center that houses 88 genomes of kinetoplastids.

As is the case for other trypanosomatids, transcription in *T. cruzi* is polycistronic, and precursor RNAs are subsequently processed into mRNAs via trans-splicing [11]. Therefore, gene expression regulation does not occur at the transcriptional level; instead, it takes place via controlling the stability and translation of specific mRNAs [12]. Indeed, lack of correlation between global transcriptomic and proteomic data has been reported [13], highlighting the importance of evaluating expression at the protein level in this parasite. Post-translational modifications also play an important role in protein function modulation by *T. cruzi*. [14]. As a result, proteomics has been recognized as a key tool to gain understanding of the global protein expression patterns in this parasite. A multitude of studies have leveraged proteomic methods to contribute to the understanding of various aspects of its biology, including developmental stage-specific protein expression, global protein content of subcellular fractions (membranes and organelles), post-translational modifications, virulence, infectivity and pathogenicity factors, metabolic pathway usage, drug resistance, as well as the parasite secretome and gene expression patterns associated with the parasite genotypes. These efforts have improved the understanding of the biology of *T. cruzi* and may help unveil mechanisms underlaying different Chagas clinical manifestations, allow the identification of novel antiparasitic drug targets and inform vaccine design.

## 3. Stage-Specific Global Protein Expression Patterns During the Parasite Life Cycle

**Figure 1:**
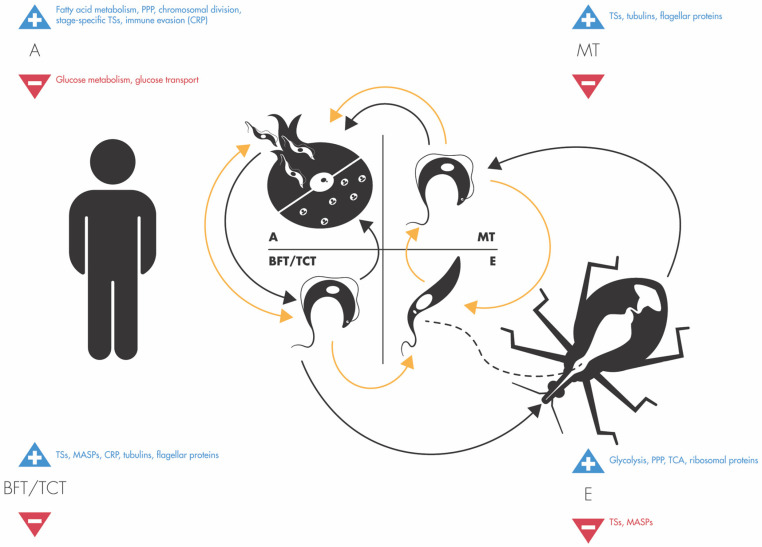
Life cycle of *T. cruzi.* The progression of *T. cruzi* through the different life cycle stages occurring in the mammalian host (**left**) and invertebrate host (**right**) is indicated by the black arrows. Orange arrows indicate the transitions that can be achieved in the laboratory using axenic or mammalian tissue culture (see text for details). Blue and red arrows indicate greater or lesser protein expression, respectively, in each life-cycle stage as reported in the reviewed proteomic studies. A = amastigote, BFT/TCT = blood form trypomastigote/tissue-culture-derived trypomastigote, E = epimastigote, MT = metacyclic trypomastigote.

*T. cruzi* possesses a digenetic life cycle, involving hematophagous triatomine bugs, commonly known as “kissing bugs”, as well as a mammalian host (including humans). The parasite transitions through morphologically different stages during its life cycle, and alternates between replicative and non-replicative forms. In the triatomine bug’s midgut, *T. cruzi* multiplies as epimastigotes, which later transform into metacyclic trypomastigotes (MTs), infectious to mammals, in the vector hindgut. Triatomines feed on the mammalian host’s blood and defecate near the bite site. MTs are present in the triatomine bug’s feces and contaminate the bite wound or mucous membranes, establishing intracellular infection in mammalian cells. The parasite multiplies in the host cell as amastigotes. After several replication rounds, amastigotes occupy most of the host cell cytoplasm and transform into trypomastigotes, which cause host cell rupture and escape into the blood stream. These free trypomastigotes infect additional mammalian host cells, disseminating the infection to other tissues. Finally, if an uninfected triatomine bug takes in blood form trypomastigotes (BFTs) up with a blood meal, the parasites will infect the bug’s digestive tract, and transform into epimastigotes in the midgut, reinitiating the cycle (Figure 1).

In addition to displaying distinct morphologies, different life-cycle forms of *T. cruzi* possess specific characteristics. For instance, only epimastigotes and amastigotes are capable of replication; the former in the triatomine vector digestive tract and the later intracellularly, in the mammalian host. Epimastigotes are unable to infect mammalian cells; however, they are infectious to triatomines. Meanwhile, trypomastigotes, both MT and BFT, are infectious to the mammalian host as well as to the triatomine vectors. Researchers have leveraged proteomic approaches to unveil protein expression patterns specific to each *T. cruzi* developmental stage, as well as during transition between stages.

A determining factor during these studies is the ability to generate *T. cruzi* populations comprised exclusively (or predominantly) of a given parasite stage, to be used as input for proteomic analysis. Different methods are available to obtain different life forms in the laboratory (Figure 1). Epimastigotes can be cultured axenically and, in stationary phase cultures, part of the parasite population spontaneously transforms into MTs. Such transformation can also be induced via nutritional stress, i.e., incubating axenic epimastigotes in low nutrient medium. MTs can then be affinity-purified from the cultures via anion-exchange chromatography on a diethylaminoethyl (DEAE)-cellulose column [15]. In contrast, the life-cycle stages present in the mammalian host must be obtained via intracellular infection. In experimentally infected animals, trypomastigotes (BFTs) egress from infected cells and reach the bloodstream, from where they can be collected. Cultured mammalian cells can be infected with MTs or BFTs, which establish intracellular infection, transform into amastigotes and multiply in the host cell cytoplasm. After ~five days of in vitro intracellular multiplication, amastigotes transform into trypomastigotes, rupture the host cell and are released into the culture medium. These are known as tissue-culture-derived trypomastigotes (TCTs) and, in general, they are thought to be equivalent to BFTs. Amastigotes can be obtained axenically, by exposing TCTs to low pH (pH 5–6), e.g., [16,17]. If MTs, TCTs, BFTs or amastigotes are cultured in axenic medium, they transform into epimastigotes and multiply. Therefore, the methods employed to obtain the parasite population to be studied via proteomics, as well as its purity, can significantly impact the experimental results.

### 3.1. Epimastigotes

Pioneering studies [14] delineated the global protein expression profile for epimastigotes. Proteins associated to metabolism, cytoskeleton assembly, protein targeting, cell cycle growth and protein synthesis were identified; tubulins, heat-shock proteins (HSPs) and prostaglandin F2-α were reported to be the most abundant proteins in this life stage. Subsequent studies reported the presence of multiple isoforms of tubulins and heat-shock proteins in this life stage [18]. Furthermore, Cordero and co-workers identified energy metabolism enzymes (glucose uptake and oxidation, i.e., glycolysis, pentose phosphate shunt or tricarboxylic acid cycle) in epimastigotes, in accordance with the high energy requirements of this proliferative stage of the parasite [19]. Additionally, epimastigotes express abundant enzymes to catalyze conversion of histidine (an abundant carbon source in the triatomine gut) into glutamate [20]. During the exponential growth phase, epimastigote cultures upregulate ribosomal proteins, consistent with the protein synthesis required for multiplication [21]. However, they have reduced expression of proteins associated with cell invasion and immune evasion (such as trans-sialidades, mucin-associated surface proteins (MASPs)) [20]. Altogether, these results indicate that epimastigotes are intensely active in terms of energetic metabolism and protein synthesis, and adapted to the use of glucose as a nutrient, along with amino acids available in the vector digestive tract. However, as expected, they display less virulence factors than mammalian-infective stages.

### 3.2. Metacyclic Trypomastigotes

Metacyclogenesis, the process by which epimastigotes transform into MTs, is of special interest, since it marks the evolution from non-infective trypomastigotes into the stage infective to mammals (including humans). Metacyclogenesis initiates in the hindgut of insect vectors, where the parasite is subjected to nutritional stress and confrontation with proteases. It has been suggested by proteomic analysis that glucose depletion leads to the elongation of the flagella, thereby increasing their area and facilitating nutrient uptake [22]. Fine characterization of protein expression patterns at different time-points of nutritional stress-induced metacyclogenesis has been attempted, identifying modulation of tubulins, some not meeting the expected molecular weights and isoelectric points in the 2D gels, suggesting varied post-translational modifications [23,24]. When reaching the stationary phase, axenically cultured epimastigotes upregulate proteins involved in autophagy (i.e., autophagy protein Apg6) [21]. Additionally, the universal minicircle sequence binding protein (UMSBP), which binds the origin of the kinetoplast replication, is acetylated in the stationary phase, despite its expression level being the same in the two growth phases, suggesting it could be a regulatory event. These changes have been suggested to mark the transition from exponential growth into metacyclogenesis [21].

Enzymes associated to the REDOX balance were found to display differential regulation in MT [24]. Additionally, cytoskeletal proteins, like actins and dyneins, were found to be upregulated in these infectious life stages, along with paraflagellar rod proteins [23]. Similar to epimastigotes, MTs express abundant enzymes to catalyze conversion of histidine into glutamate [20]. However, as opposed to epimastigotes, MTs must be equipped to face the mammalian immune system, and they express larger quantities of proteins involved in antioxidant defenses [20]. A large proportion of proteins identified in MTs are surface proteins (35 members of the trans-sialidase superfamily, one mucin, and one GP63 protease) involved in host–parasite interplay (evasion from the host immune response, invasion of mammalian cells and glycosylation of parasite surface molecules) [19].

It has also been shown that MTs have the ability to transform back into epimastigotes [25]. MTs incubated for 72 h liver infusion tryptose blood medium display a global protein expression pattern resembling that of epimastigotes, including downregulation of virulence factors, i.e., proteins related to parasite infectivity, adhesion and invasion, immune evasion and response to oxidative stress. These intermediary forms, termed “recently differentiated epimastigotes” (rdEpis) are infectious to cultured cells and experimental animals, and they are resistant to complement (a property regular epimastigotes do not have and which makes them not infectious in the human host).

### 3.3. Amastigotes

Amastigotes are intracellular replicative forms, which multiply inside mammalian host cells. Early proteomic studies showed that amastigotes display low expression of glucose transporters, and abundant expression of enzymes associated to fatty-acid oxidation into acetylCoA (AcoA), as well as subsequent AcoA oxidation [20]. As expected in a replicative form, proteins involved in chromosomal division are expressed by amastigotes along with enzymes involved in the oxidative phase of the pentose phosphate pathway, which generates precursors for nucleotide biosynthesis [26].

Proteomic studies have shown that amastigotes display stage-specific surface proteins, including trans-sialidases and other glycoproteins, likely stage-specific antigens [26]. A putative thymet oligopeptidase (TOP), which could potentially participate in degradation of proteasome-derived peptides by interfering with MHCI antigen presentation, was found to be overrepresented in amastigotes. Additionally, immune escape-related proteins, like complement regulatory protein and calreticulin, which promote parasite resistance to complement-mediated lysis, were found to be highly upregulated in amastigotes [16,25].

### 3.4. Blood Form Trypomastigotes/Tissue-Culture-Derived Trypomastigotes (BFT/TCTs)

In comparison to epimastigotes, TCTs were found to produce larger amounts of trans-sialidases and mucin-associated surface proteins (MASPs), surface proteins associated with cell invasion and immune evasion [20,27]. Additionally, trypomastigotes upregulate the production of complement regulatory proteins to prevent lysis by host complement [25].

The cell surface subproteome of TCTs has been reported to be largely similar to that of axenic amastigotes [16]. Stage-specific surface proteins identified corresponded mainly to trans-sialidases and glycoproteins, likely stage-specific antigens, though authors pointed out some of these proteins may not necessarily be associated with the plasma membrane and may also correspond to microvesicles/exosomes. However, greater expression of proteins related to flagellar motility, including paraflagellar rod components and tubulins, has been reported in TCTs compared to axenic (pH-induced) amastigotes and epimastigotes [26,28]. Additionally, metabolic enzymes and heat-shock proteins, among others, are differentially expressed between TCTs and amastigotes [28].

## 4. Subcellular Proteomes

Proteomics in combination with cell fractionation approaches have been employed to explore the protein components of different subcellular compartments in *T. cruzi* cells. It has been pointed out that an important limitation of such strategy is the inability to generate completely “pure” organelles or subcellular fractions and that, in conjunction with the high sensitivity of the proteomic methods, this creates the possibility of incorrect assignment of proteins’ location [29]. Individual proteins identified as part of proteomes from purified organelles or subcellular fractions must, therefore, be considered only as candidates for a particular subcellular location, which must be experimentally confirmed. In some cases, researchers have confirmed predicted subcellular locations for selected subsets of the proteins of interest [30,31] via expression of recombinant proteins and microscopy.

Another limitation is that proteomic studies regarding subcellular localization of *T. cruzi* proteins have largely concentrated in epimastigotes. One important exception is Won and co-workers, who performed proteomic analysis of both epimastigotes and amastigotes and confirmed the location of a subset of proteins to the flagellum of both these parasite stages as well [31].

Early studies [29] analyzed the proteins in the organellar cell fraction of *T. cruzi* epimastigotes. A subset of these proteins was selected and their gene copy number, sequence variation, transmembrane domains and targeting signals were characterized. Importantly, the authors cloned the genes and expressed the corresponding proteins with a c-myc epitope tag in *T. cruzi* epimastigotes. Immunofluorescence microscopy revealed the localization of these proteins in different cellular compartments, such as endoplasmic reticulum, acidocalcisome, mitochondrion and putative cytoplasmic transport or delivery vesicles. Additional studies, which have concentrated on specific organelles or macromolecular complexes, are reviewed below.

### 4.1. Ribosomes

Ayub and co-workers purified *T. cruzi* ribosomes and analyzed them via mass spectrometry to characterize their proteome. *T. cruzi* ribosomal proteins were shown to have 50% sequence identity with yeast ribosomal proteins, although they are longer because of the presence of N- and C- terminal extensions unique to trypanosomatids. Importantly, *T. cruzi* 60S subunit proteins L22 and L44 were detected for the first time in this study [32].

### 4.2. Reservosomes

Epimastigotes obtain proteins and lipids from the culture medium by endocytosis and store them in bodies known as reservosomes, which contain secretory-system-derived proteolytic enzymes. Therefore, reservosomes are sites for both nutrient storage and degradation [33]. To characterize their molecular signature, Sant’Anna and co-workers purified epimastigote reservosomes by differential centrifugation, and analyzed their membrane-associated components via mass spectroscopy. The reservosome membrane fraction was found to be rich in proteins associated to hydrolase activity, carrier and ion-transport activities, nucleotide-binding, transferase activity, ion-binding and oxidoreductase activity. A large number of reservosomal proteins were related to metabolism. Proteins included enzymes and proton pumps, small GTPases of the Rab family, ABC transporters, lysosomal hydrolases and P-type H+-ATPase (TcHA3). Cruzipain, the major *T. cruzi* protease, was also detected. In this study, however, experimental confirmation of intracellular location was not pursued.

### 4.3. Contractile Vacuole

Contractile vacuoles are specialized organelles, which play a key role in allowing protists to maintain cell volume [34]. During its life cycle, *T. cruzi* develops in different environments, in some of which it encounters osmotic stress (vector digestive tract, mammalian kidney), and its contractile vacuole is critical to maintain cell-volume stability [35]. Ulrich and co-workers purified epimastigote contractile vacuole fractions using differential gradients and centrifugation. A large number (39) of members of the dispersed gene family (DFG-1) proteins, calpain-like cysteine peptidases, amastins (transmembrane glycoproteins characteristic of amastigotes), transport-related and intracellular proteins like small G-proteins (Rabs), soluble N-ethylmaleimide-sensitive factor (NSF), adaptor protein (SNAP) receptors (SNAREs), in addition to transporters and channels, among other proteins, were identified in these fractions [30]. A subset of these proteins was expressed with GFP tags and verified via fluorescence microscopy, including Rab11, Rab32, AP180, ATPase subunit B, VAMP1 and phosphate transporter, which predominantly localized to the vacuole bladder [30].

### 4.4. Plasma Membrane Fractions

Queiroz and co-workers studied plasma-membrane-associated proteins by two complementary methods: trypsinization (shave) of intact life cells and biotinylation of cell surface followed by affinity isolation with streptavidin columns. A control sample where no treatment was performed was included to account for secreted proteins. The resulting proteins were analyzed by LC-MS/MS. Authors concluded that both methods allowed characterization of plasma membrane proteins, in a complementary manner, and suggested they must be used in conjunction [36].

### 4.5. Nucleus

Proteomic approaches have been employed to explore the nuclear subproteome of *T. cruzi* by isolation of the nuclear fraction employing a sucrose gradient, centrifugation coupled with proteomic analysis [37]. LC-MS/MS identified 864 proteins; approximately one-third of them were previously uncharacterized and one-third had not previously reported via proteomic analysis. As expected, proteins identified were related to chromatin organization and DNA-binding functions, including centrins, histones, nucleolar proteins, nucleosome assembly proteins, ribosome proteins, transcription factors and structural nuclear proteins. Additionally, proteins involved in RNA processing were identified, including poly-A binding protein, RNA helicases, elongation factors and eukaryotic translation initiation factors. Additionally, 53 ribosomal proteins were identified, 25 of which were components of the 40S ribosomal subunit and the other 28 as components of the 60S subunit. Ribosome biosynthesis occurs primarily in the nucleoli and nucleoplasm and, therefore, detection of ribosomal proteins in the nuclear fraction is not surprising.

### 4.6. Glycosomes

Glycosomes are specialized *T. cruzi* organelles, related to peroxisomes [38]. As opposed to other eukaryotes, in which glycolysis occurs in the cytoplasm, the initial seven glycolytic reactions in *T. cruzi* occur in glycosomes, which contain enzymes associated with glycolysis and other central carbon metabolism pathways [38]. Several other metabolic reactions key for β-oxidation of fatty acids, purine salvage, pentose phosphate pathway, gluconeogenesis and biosynthesis of ether lipids, isoprenoids, sterols and pyrimidines take place in glycosomes [39]. The same authors found other enzymes for glycolysis, oxidative and non-oxidative branches of the pentose phosphate pathway (PPP) sugar nucleotide synthesis, ether lipid biosynthesis, lipid metabolism, purine and pyrimidine synthesis, sterol synthesis, response to oxidative stress, gluconeogenesis, membrane transporters. These data allowed the authors to confirm the presence of enzymes involved in these various metabolic pathways in the parasite’s glycosomes. However, no experimental confirmation of protein localization was pursued in this study [39].

### 4.7. Spliceosomes

As opposed to most eukaryotes, trypanosomes transcribe protein-coding genes into polycistronic RNA, which subsequently is processed into individual RNAs via trans-splicing. During this process, 5′splice-leader (39-bp in length) RNAis added to each individual RNA in the polycistronic unit [40]. In order to characterize the proteins forming part of *T. cruzi* spliceosomes, Barbosa and co-workers expressed *T. cruzi* TAP-tagged spliceosome-associated proteins TcCwc21 and TcSmD1, and used them to affinity-purify other protein components of the spliceosomal macromolecular complex and subsequently identify them via mass spectrometry. A total of 115 proteins were identified, which have conserved orthologs of *T. brucei* and/or human spliceosomes, especially the U5-snRNP related, Sm/LSm proteins and helicases. A core set of protein components common to human and *T. brucei* spliceosomes was identified, namely SNRNP200, SNRNP116, PRPF8, HSPA8, SNRPD1, SNRPB and SNRPD2. Authors highlight the fact that these similarities between human and trypanosomatid spliceosomes indicate that molecular complexes carrying out cis- and trans-splicing are mostly similar.

### 4.8. Flagellum

The *T. cruzi* flagellum is composed of nine pairs of peripheral microtubules and two central microtubules [41]. Motor functions are attributed to this organelle; however, the distal end is a recognized domain of signaling of the environment [31]. In an elegant study, the same authors used the biotin ligase TurboID to attain proximity-dependent biotinylation of flagellar proteins in *T. cruzi* epimastigotes and amastigotes. These authors employed CRISPR/Cas-9 facilitated association of the TurboID ligase to the small myristoylated protein 1-1 (TcSMP1-1), which they had showed to localize to the amastigote flagellum in a previous study [42]. Using this approach, 218 candidate flagellar proteins in epimastigotes and 99 in amastigotes were identified, forty of which were common to the two developmental stages. The candidate protein list was shown to be enriched in orthologs to other trypanosomatid species for which a flagellar localization had been annotated. The authors then successfully validated the location of three of the candidate proteins into the parasite flagellum, namely calpain 1.3, cellular apoptosis susceptibility protein 3 (CARP3) and a hypothetical protein, for which they expressed epitope-tagged versions that could be visualized in fixed parasites by fluorescence microscopy.

## 5. *T. cruzi* Intra-Specific Diversity and Proteome

*T. cruzi* displays high genetic diversity with clearly structured genetic subpopulations. The parasite is believed to reproduce in primarily clonal fashion, with occasional episodes of hybridization/genetic exchange [43,44,45,46], although the possibility of more frequent genetic exchange has also been proposed. Based on genomic and mitochondrial DNA, six major genetic lineages have been identified, which are designated as “discrete typing units” (DTUs) TcI-TcVI [47,48]. Subsequently, an additional genetic lineage, Tcbat, was also described [49].

Most reports about *T. cruzi* proteomics in the literature analyze exclusively a single parasite strain or isolate. In the instances where more than one strain is analyzed, differences between strains become evident [50,51]. Among the small number of reports studying more than one parasite stock, Tavares de Oliveira, in 2018, reported differences across the proteomic patterns yielded by two parasite isolates from chagasic patients, one corresponding to DTU TcI and the other to TcII [52]. Similarly, distinct proteomic patterns were displayed by different isolates derived from triatomine bugs [53].

In an effort to examine the entire breath of *T. cruzi* diversity, Telleria and co-workers studied axenically cultured epimastigotes belonging to DTU TcI-TcVI, and found that the phylogenetic structure resulting from proteomic (2DE) analysis is highly correlated with that obtained by genetic marker analysis. Proteins like α-tubulin, β-tubulin, HSP85-like protein, elongation factor 2 and iron superoxide dismutase displayed DTU-specific expression patterns [54]. The authors observed various spots corresponding to tubulins and other proteins, which might result from post-translational modifications.

Interestingly, axenic cultures of *T. cruzi* epimastigotes containing a mix of parasites from different DTUs display global proteomic profiles different from those of the individual strains, indicating that expression of a subset of proteins can be potentiated or inhibited when strains are mixed [55]. The significance of mixed *T. cruzi* infections in vectors or human hosts is not understood. However, these experimental results suggest a possible communication equivalent to a molecular dialogue between genotypes. It is suggested that expression levels of certain proteins can be potentiated or inhibited when strains coexist [55].

Taken together, these results highlight the importance of strain and DTU choice during proteomic studies about *T. cruzi*. Care must be exercised when generalizing findings, because strain choice can significantly impact experimental results. Additionally, when analyzed together, parasites from different strains or DTUs can impact each other’s global protein expression. The implications of the latter phenomenon in the infection of vectors and mammalian hosts remain unknown.

## 6. Parasite Pathogenicity Factors

As already mentioned, *T. cruzi* is a highly variable pathogen, and parasite strains differ greatly in terms of their infectivity and pathogenicity. Meanwhile, the molecular factors that determine pathogenicity and the clinical manifestations of Chagas disease are not totally clear. Therefore, a common strategy employed by researchers has been to characterize strains with different infectivity or pathogenicity via proteomics, in hopes to identify proteomic signatures or individual proteins expressed exclusively or primarily by pathogenic strains.

Some studies have compared strains with different pathogenicity within the same DTU [51]. Dias, in 2011, explored the expression pattern of two TcI strains, one isolated from acute infection and one from cardiac form chronic patients. They identified tryparedoxin peroxidase, lipoamide dehydrogenase, tyrosine amino transferase and HSP70 as overexpressed proteins in the acute Chagas isolate. Dias and co-workers and Zago and co-workers performed proteomic profiling of three TcI isolates (2DE-MALDI-TOF), two displaying higher parasitemia and larger chronic damages than the other. Proteins associated with cell invasion (e.g., cysteine peptidases, surface glycoprotein gp82 and cruzipain), cytoskeletal dynamics and motility (isoforms of actin and tubulin, cofilin-1, KMP11 and paraflagellar rod proteins) were enriched in epimastigotes and trypomastigote stages of the pathogenic strain [51,56]. Furthermore, proteins involved in redox homeostasis like tryparedoxin peroxidases, peroxiredoxins, thioredoxin-dependent peroxide reductase and iron superoxide dismutases were overexpressed in the trypomastigotes of the most pathogenic strains. The expression of several proteins related to redox homeostasis was confirmed experimentally. The authors suggested that the overexpression of these proteins constitutes a virulence factor in trypomastigotes of TcI strains.

Other studies have compared strains belonging to different DTUs [57]. These authors compared the VFRA strain (TcI), which produces chronic infection in mice, with the Y strain (TcII), which causes lethal acute infections in the mouse model, along with higher parasitemia and higher replication rates in mouse macrophages and in the heart tissue. Overall, despite belonging to different DTUs, the proteomes of both strains were found to be similar. The authors suggested that the differences in pathogenicity are correlated with higher production of antioxidant defenses in the VFRA strain and higher expression of proteins associated with parasite replication in the Y strain.

Finally, in what probably constitutes the best study of this kind, San Francisco and co-workers compared two clones derived from the same parental strain (H510 C8C3, TcI) with different pathogenicity. For 30 years, one of the clones (virulent) was passaged weekly in mice, while the other (low virulence) was passaged as axenic epimastigotes. This resulted in the selection of two polar phenotypes: the virulent clone produced higher parasitemia and parasite load in tissues (heart, liver, quadriceps and lung) in mice, and significantly higher infectivity over cultured cells. The proteomic analysis showed biological pathways enriched in the virulent strain were associated with bioenergetics and biosynthetic pathways (including the TCA cycle and citrate metabolism), along with virulence factors such as redoxins, cruzipain, trans-sialidases, complement resistance protein and Tc-85. Therefore, virulence would depend on increased capacity to generate energy output, upregulation of virulence factors and greater capacity to face oxidative stress [58].

## 7. *T. cruzi* Secretome

*T. cruzi* has been shown to secrete proteins in large (>100–200 nm) plasma-membrane-derived vesicles, as well as smaller vesicles (<100 nm), derived from microvesicular bodies in the flagellar pocket region. Additionally, free proteins without association with vesicles are released by the parasite [59].

Secretomes have been characterized for various developmental forms of *T. cruzi*, including epimastigotes [60], axenic amastigotes [61], trypomastigotes [62] and MTs [60]. Proteins released into the culture medium, either free or associated to vesicles, have been analyzed via mass spectrometry and qualitative differences have been identified between secretomes across parasite stages. For example, proteomic analysis of extracellular vesicles (EVs) indicates an expansion of trans-sialidases in trypomastigotes as compared to epimastigotes [62]. Mandacaru, in 2019, found an increased number of mucins and mucin-associated proteins during amastigogenesis, while the number of released trans-sialidases and surface gp63 proteases was reduced [60]. Egress of intracellular *T. cruzi* forms (amastigotes and trypomastigotes) upon host cell rupture at the end of the intracellular cycle is accompanied by the release of hundreds of parasite-derived proteins, most of which belong to multigenic families, such as trans-sialidase and trans-sialidase-like proteins, gp63 surface proteins, MASPs and DGF1 [50,61]. Together, these results suggest that protein secretion by *T. cruzi* takes place throughout the parasite’s life cycle, tailored to the different environments the parasite must confront.

Additionally, differences in the number and composition of secretory vesicles have been reported to exist between parasite strains belonging to the TcI and TcII lineages [63]. These authors reported TCT from a TcI strain released a higher number of vesicles containing trans-sialidases and cruzipain (implicated in pathogenesis and immunopathology) than the TcII strain. The authors tested the hypothesis that the secreted proteins could modulate parasite–host interactions, by pre-treatment of tissue-cultured cells with vesicles prior to in vitro infection with the parasite. They determined that exposure to the vesicles modulated infection and parasite multiplication in vitro. Although, in our opinion, care should be exercised when extrapolating findings from a single strain to an entire DTU, these findings highlight that secretomes are not uniform among parasites from different strains and secreted proteins can impact cell infection and parasite multiplication. In the context of a highly variable parasite, such as *T. cruzi*, this adds a layer of complexity to host–parasite interactions during infection.

Characterization of the *T. cruzi* secretome has been largely performed using in vitro systems; however, characterization of parasite-derived extracellular vesicles has been attempted in vivo as well. Cortes-Serra, in 2020, studied EVs present in the serum of a chronic *T. cruzi*-infected patient, whose infection reactivated due to immunosuppression before heart transplant. They identified *T. cruzi* pyruvate phosphate dikinase (PPDK), a glycosomal enzyme, among the vesicular contents [64], although the role this protein may play during infection remains unknown. Studies involving larger patient cohorts are warranted.

Additionally, qualitative and quantitative variations of secreted proteins between strains might be related to infectiveness and evasion of the host mammalian immune response. The *T. cruzi* secretome represents a diverse assortment of proteins involved in parasite metabolism, signaling and nucleic acid binding, which might have a direct role in parasite survival and virulence [60].

## 8. Post-Translational Modifications

Post-translational modification is thought to play a key role in protein function regulation in *T. cruzi*. Phosphorylation, glycosylation, acetylation, nitrosylation, sumoylation in *T. cruzi* have been explored via proteomic approaches.

### 8.1. Chromatin Modifications

Chromatin is packed in nucleosomes, composed of two units of each core histone (H2A, H2B, H3 and H4) around which 146 bp of DNA are coiled around. Meanwhile, H1 links nucleosomes together [65]. Histone post-translational modifications are crucial because they impact chromatin structure, and, consequently, gene expression, modulating key cellular processes. Much has been learned through proteomic analysis about histone post-translational modification of histones. De Jesus and co-workers extracted chromatin from epimastigotes and MTs and used high-resolution mass spectrometry to identify post-translational modifications, detecting 44 novel modifications, including acetylations (18), monomethylations (7), dimethylations (8), trimethylation (7) and phosphorylations (4). These authors found that epimastigotes display more histone modifications than trypomastigotes, and reported several novel types of modifications [66].

Although the role of histone nitrosylation in *T. cruzi* remains to be determined, histone nitrosylation in H2B and H3 on Cys64 and Cys126 has also been described [67,68]. These authors employed chromatin immunoprecipitation (ChIP) with an anti-nitrotyrosine antibody to purify nitrated proteins, which were subsequently analyzed by mass spectrometry. Histones H1, H2B, H2A and H3 were identified to be some of the most common nitrated DNA-binding proteins. In addition, they reported an increase in histones H1, H2B, H2A and H4 nitration levels when parasites were incubated with extracellular matrix, with which trypomastigotes interact in the contexts of host cell invasion. These results indicate that interaction with the extracellular matrix (ECM) influences chromatin structure. Additionally, [69] detected previously known acetylation sites, K10 and K14, in H4, as well as K7, K15 and K17 in H2B.v, reported a new H3 acetylation site at position K79.t. and [70] identified methylated sites in different histones: H4R90me2, H4R54me2, H3.VK95me3, H2BK97me, H1K10me2, H2BR61me, H2BR61me2 and H2AK102me2.

Histone deacetylases (HDACs) remove acetyl groups from histones, modulating chromatin compaction and, consequently, gene expression. De Oliveira de Santos et al. (2019) studied the effects of trichostatin A (TSA), an HDAC inhibitor, over histone acetylation in *T. cruzi* by quantitation of acetylated histones via LC-MS/MS [71]. Quantitative proteomic analyses indicated an increase in histone acetylation 72 h post-TSA treatment. The major effects on *T. cruzi* were not found to take place via histones, but rather via microtubule cytoskeleton dynamics and impaired kDNA segregation, resulting in polynucleated parasites with altered morphology.

### 8.2. Nitrosylation

As mentioned above, early in the process of cell invasion, *T. cruzi* trypomastigotes are known to interact with the ECM; a complex structure of more than 300 proteins and glycoproteins [72]. Using resin-assisted enrichment of thiols combined with mass spectrometry [68] mapped S-nitrosylated proteins from *T. cruzi*, and decrease in S-nitrosylation upon contact with the ECM was detected. S-nitrosylated proteins were found to be enriched in the ribosome. Additionally, proteins related to transport, carbohydrate (glycolysis/TCA cycle), lipid metabolism, gene transcription (histones), proteases and cytoskeleton remodeling, indicating that profound adjustments of the parasite physiology in response to ECM occur before host cell infection.

### 8.3. Phosphorylation

As in other organisms, reversible phosphorylation is believed to play a key role during signaling in *T. cruzi*. Two percent of this parasite’s genome encodes kinases [73]. Proteomic approaches have been harnessed to gain insights into the role of phosphorylation in global cell signaling networks in *T. cruzi.* The authors of [74] attempted to generate a comprehensive phosphorylation map for *T. cruzi* epimastigotes via liquid LC-tandem MS, identifying 237 phospho-peptides in 199 individual proteins, along with mapping of 220 phosphorylation sites (including on serine, threonine and tyrosine). The existence of tyrosine-phosphorylated proteins was confirmed via Western blot. Phosphorylated proteins were involved in motility, transportation, metabolism (glycolytic enzymes), pathogenesis (trans-sialidases and dispersed gene family protein 1), nucleic acid dynamics and signaling. Importantly, phosphorylation of histone 2B was reported by these authors. Additionally, proteomic methods have been used to identify phosphorylation in serine 23 of histone 2B in *T. cruzi* [66]. Additional studies have applied super-SILAC (super-stable isotope labeling by amino acids in cell culture) and LC-MS/MS to characterize global protein phosphorylation patterns during nutritional stress-induced metacyclogenesis in *T. cruzi* [75], identifying 4205 protein groups and 3643 phosphopeptides, and locating 4846 phosphorylation sites. Phospho-sites modulated during metacyclogenesis were located in proteins associated with fatty acid synthesis, regulation of protein expression, structural components, stress response and intracellular signaling, suggesting that phosphorylation/dephosphorylation events trigger metayclogenesis.

### 8.4. Glycosylation

Mass-spectrometry-based glycoprofiling analysis of epimastigotes and trypomastigotes has been performed [76], identifying 1306 N-glycosylation sites in 690 *T. cruzi* proteins. This has allowed for detailed characterization of the N-linked and O-linked glycoproteome, and unveiled stage-specific glycoproteomic signatures, in terms of epimastigote- vs. trypomastigote-exclusive N-linked and O-linked glycopeptides. Greater glycosylation was identified in gp85/trans-sialidase, MASP, mucins and GP63 family proteins in trypomastigotes. Additionally, [77] employed proteomics to show that O-GlcNAcylation, a monosaccharide post-translational modification of serine and threonine residues, takes place in *T. cruzi.* Mass spectrometry allowed for the identification of 1271 putative O-GlcNAcylated proteins and six modification sequences. O-GlcNAcylation was found in clathrins, RNA helicases, DNA polymerases, trans-sialidases and uncharacterized proteins. This post-translational modification is believed to allow for protein function regulation in a similar fashion to phosphorylation.

### 8.5. Methylation

De Almeida, in 2023, applied mass-spectrometry-based proteomics (LC-MS/MS) to describe non-histone methylation in epimastigotes. This post-translational modification takes place in arginines and lysines. A total of 1253 methyl sites in 824 methylated proteins were identified in proteins involved in translation, RNA and DNA binding, amino acid and carbohydrate metabolism [70].

### 8.6. Non-Histone Acetylation

Moretti, in 2018, identified 89 ε-lysine-acetylated sites in 235 proteins in epimastigotes. The lysine-acetylated protein set is enriched in enzymes involved in oxidation/reduction balance, crucial for parasite survival. Furthermore, the eukaryotic translation initiation factor 5A (eIF5A) was determined to have an acetylation at lysine 50 (K50). It was proposed by the authors that different acetylation profiles could correlate with parasite environmental fitness. Therefore, the data they presented can be used to determine experimentally the role of each identified modification. Finally, they proposed that development of specific acetyltransferase and deacetylase inhibitors can potentially lead to the discovery of new treatment options for Chagas disease [69].

### 8.7. SUMOylation

The small ubiquitin-like modifier pathway (SUMO) is a post-translational modification, which modulates protein activity, stability and/or localization. [78] used bioinformatics to identify the existence of orthologous genes for the SUMOylation pathway in *T. cruzi* and confirmed this system is active in the four major life-cycle stages of the parasite via Western blot analysis of total parasite homogenates using anti-TcSUMO polyclonal antibodies. Additionally, these authors generated epimastigote lines expressing a double- tagged *T. cruzi* SUMO, and SUMOylated proteins were isolated by affinity chromatography. By two-dimensional liquid chromatography–mass spectrometry, a total of 236 proteins with diverse biological functions were identified as potential *T. cruzi* SUMO targets. Metacaspase-3 was experimentally validated as a SUMOylation substrate.

### 8.8. Pathway Crosstalk

Finally, it is important to consider that these post-translational modifications coexist in the parasite cells, and that they can potentially overlap and influence each other. For example, nitrosylation and phosphorylation were determined to occur in both kinases and phosphatases, suggesting crosstalk between them [68]. In addition, 171 *T. cruzi* proteins found to be methylated were previously reported to contain phosphorylation sites, including flagellar proteins and RNA-binding proteins, indicating that there may be an interplay between these different modifications in non-histone proteins [70].

## 9. Interactomes

Another strategy used by researchers to probe *T. cruzi* biology has been to employ mass spectrometry methods to identify the proteins specifically interacting with a given molecule, molecules, or macromolecular complex of interest. This has been of special utility when characterizing the REDOX system and post-translational mechanisms in the parasite, characterizing chromatin-associated proteins, as well as the composition of the macromolecular complexes associated with parasite mRNA translation.

### 9.1. Enzymes Related to Oxidative Stress Response

Throughout the life cycle, *T. cruzi* is exposed to oxidative stress imposed by reactive oxygen species (ROS), such as superoxide anions, hydrogen peroxide (H_2_O_2_) and hydroxyl radicals, derived from its own metabolism, host cells and external agents [79]. An imbalance between the production of ROS and the antioxidant defense system could be lethal to the parasite [79]. The parasite’s antioxidant mechanism is based on the combined action of several molecules, such as tryparredoxin peroxidases (TcTXN1 y II), which tackle oxygen free radicals and hydrogen peroxide through peroxidase activity, and peroxynitrite reduction [80]. These molecules, in conjunction with trypanothione reductase, have functions similar to glutathione in mammalian cells, i.e., intervening in redox reactions dependent on NADPH [80]. Trypanothione is a thiol that gives up electrons for detoxification of hydroperoxides [81]. The ability of the parasite to manage ROS production and antioxidant capacity is crucial to its survival.

Interactomes of tryparedoxin 1 (TcTXN1) [82] and 2 (TcTXNII) [83], as well as the mitochondrial tryparedoxin reductase (TcMPx) [84], have been reported. Piñeyro and co-workers designed a 6-His-tagged TcTXN1 active site mutant lacking the resolving cysteine, which causes the enzyme to form heterodisulfide complexes with potential substrates, and expressed it in *T. cruzi* epimastigotes. The TcTXN1 complexes present in parasite lysates could then be affinity-purified thanks to the His-tag and subsequently analyzed by 2DE/MS. The strategy was validated, showing the interaction of the recombinant protein and cytosolic peroxiredoxin. This approach allowed the identification of 15 putative TcTXN1 interacting proteins, whose functions are related to oxidative metabolism and protein metabolism (synthesis, ubiquitination, degradation). A similar approach was employed by [83] to explore the interactome of TcTXNII. They expressed a recombinant TcTXNII lacking the resolving cysteine in the active site, expressed it, incubated it with parasite lysates and identified putative TcTXNII proteins separated in SDS-PAGE gels. Sixteen putative proteins were identified, which are involved in the antioxidant system, energy metabolism, cytoskeleton and protein translation. Finally, the interactome for mitochondrial tryparedoxin peroxidase (TcMPx) was explored under physiological and oxidative stress conditions (i.e., the latter involves exposing parasites to hydrogen peroxide) [84]. Tagged recombinant TcMPx was expressed in epimastigotes, parasites were lysed and the TcMPx interactions were explored via 2DE coupled to mass spectrometry. Under physiologic conditions, the identified proteins had functions related to oxidoreductase activity and stress response. Meanwhile, under oxidative stress conditions, identified proteins were involved in amino acid metabolism, processes related to microtubules, stress responses and protein polymerization.

### 9.2. Chromatin

Leandro de Jesus and co-workers purified chromatin from epimastigotes and trypomastigotes and studied associated proteins via high-resolution mass spectrometry. Micrococcal nuclease digestion showed both life stages have equivalent nucleosome contents. However, epimastigote chromatin contains a much larger diversity and number of associated proteins than that of trypomastigotes [85]. Additionally, proteins involved in DNA replication, such as proliferating cell nuclear antigen (PCNA), replication protein A (RPA) and DNA topoisomerases were detected only in epimastigote chromatin. Ribosomal proteins were found to be enriched in epimastigotes, which is also expected, because RNA maturation occurs mainly at the nuclear space in trypanosomes [86]. The number and diversity of proteins associated with trypomastigotes were much lower (1/3 of their chromatin was found to be histones in contrast to 1/20 in epimastigotes). Importantly, the differential expression of seven selected proteins was confirmed experimentally, via Western blot and chromatin immunoprecipitation (ChiP). Although proteins involved in the cytoskeleton, ribosome and mitochondria and metabolic processes were also detected, their role must be verified to rule out contamination.

### 9.3. Polysomes

In a study carried out by Alves and co-workers, epimastigote polysomes (groups of ribosomes joined to a single mRNA molecule) were separated into glucose gradient, either during the parasite growth phase or under nutritional stress. As expected, polysomes in growing parasites were much longer, containing up to several ribosomes, while in stressed parasites, polysomes were much shorter and less abundant. The larger percentage of proteins identified were ribosomal proteins (70% for growing and 59% for stressed parasites). Non-ribosomal proteins included RNA-binding proteins, cytoskeletal components, heat-shock response proteins and proteins related to metabolism and translation. Surprisingly, metabolic proteins were the largest group of non-ribosomal proteins. The authors suggest this might be due to multifunctional enzymes playing roles during translation [87].

Wippel and co-workers studied the interactome of the RNA-binding protein-9/ribonucleoprotein (RBP9-mRNP) complex. This complex is of interest because of the lack of transcriptional regulation in *T. cruzi* genes. RNA-binding proteins associate with mRNAs and others to form ribonucleoprotein complexes (mRNPs), which are crucial in post-transcriptional regulation. As part of the characterization of *T. cruzi* RBP9, an RNA-binding protein unique to trypanosomatids, the authors determined it is located in the cytoplasm in epimastigotes, that it is expressed during metacyclogenesis but not in MTs and that it is located in cytoplasmic translational complexes. They employed a proteomic shotgun approach to characterize the proteins interacting with RBP9. In growing epimastigotes, RBP9 interacts with proteins involved in RNA metabolism, namely ZC3H39, PABP1/2, ALBA3/4 and UBP1/2 and ribosomal proteins. Authors suggest RBP9 is part of mRNP complexes in the cytoplasm involved in mRNA translation regulation [88].

### 9.4. Inositol Phosphate Interactome

Mantilla and co-workers used an affinity-based approach to identify novel targets of inositol heptakis-phosphate (5-IP_7_), a high-energy metabolite implicated in phosphorylation that regulates multiple cellular processes. Pull-down assays with immobilized 5-IP_7_ coupled with mass spectrometry in *T. cruzi* epimastigotes and amastigotes were conducted [89]. The proteins identified suggest that 5-IP_7_ participates in various metabolic and signaling pathways in epimastigotes and amastigotes, including ribosome biogenesis, and trafficking processes associated to the contractile vacuole.

## 10. Response to Compounds, Drug Resistance

Proteomic approaches have also been leveraged to identify characteristics that make the parasite resistant to the drugs available for treatment, including benznidazole [8,90], as well as the parasite response to other compounds, including quinone derivatives and naphthoimidazoles derived from β-lapachone piplartine, synthetic diamines and organometallic compounds [13,91,92,93,94]. A common theme in these studies is the modulation of proteins involved in antioxidative responses [8,13,91,92,93,94].

## 11. Perspectives and Conclusions

Proteomic approaches have played an important role in understanding key aspects of the biology of *T. cruzi*, and hold the potential to shed light over additional, not yet understood, fundamental features of parasite/host interplay during Chagas disease. The parasite’s particularities regarding gene expression regulation (i.e., lack of transcriptional regulation) limit the applicability of transcriptomics and make proteomics even more valuable for studying the biology of *T. cruzi*.

Core protein expression features of the different life-cycle forms of *T. cruzi* have been described via proteomic methods. These studies have also shown that our understanding of the intermediary forms that may mediate stage transitions is still limited and needs to be further explored and more clearly defined.

Furthermore, subcellular proteome analysis has provided a window into organelle and macromolecular complex (e.g., polysome) composition and function in *T. cruzi*. However, complete, contamination-free, organelle or molecular complex purification is unattainable. This fact, coupled with the high sensitivity of mass spectrometry methods, creates the possibility of erroneous cellular location assignment for proteins. Therefore, experimental validation (e.g., immunofluorescent or electronic microscopy, chip, etc.) is essential to confirm putative location assignment obtained by proteomic methods. Although individual validation of each protein of interest is not possible, validation of at least some selected proteins is desirable. Such validation is missing in many proteomic studies dealing with *T. cruzi*.

The broad genetic diversity of *T. cruzi* is reflected in varying global protein expression patterns displayed by DTUs and strains. The choice of the strain to be employed in proteomic studies must, therefore, not be taken lightly. Additionally, most published proteomic studies are limited to a single *T. cruzi* strain. Although it might be impossible or impractical to include a large enough number of strains to represent the intra- and inter-DTU variability of the parasite, it must be emphasized that extrapolation of findings to other DTUs, and even to strains within the same DTU, must be carried out with caution. Ideally, experimental validation would be performed.

When comparing global proteomic patterns in pathogenic vs. non-pathogenic strains, the use of phenotypically different clones derived from the same parental strain is ideal. In our opinion, this constitutes the best experimental strategy reported in the literature so far for the purpose of contrasting proteomic patterns with the aim of identifying pathogenicity determinants, since it eliminates confounding factors such as phylogenetic differences between strains. However, widespread implementation of this strategy is prevented by the unavailability of such strain pairs. Importantly, it would be desirable to extend the findings from pathogenic vs. non-pathogenic strain pairs to a variety of different strains, validating the proteomic patterns across the entire breath of *T. cruzi* genetic diversity.

Finally, proteomic datasets offer new opportunities for analysis of protein localization and functionality, allowing the unraveling of poorly characterized parasite/host interactions. Proteomic data repositories open up the opportunity for in silico exploration, which might shed further light on fundamental parasite biology, and inform drug design and vaccine development.

## Data Availability

No new data were created or analyzed in this study. Data sharing is not applicable to this article.
